# Facilitators and barriers to adhere to monitoring disease activity with ePROs: a focus group study in patients with inflammatory arthritis

**DOI:** 10.1007/s00296-022-05263-5

**Published:** 2023-01-10

**Authors:** Bart F. Seppen, Jimmy Wiegel, Michael T. Nurmohamed, Wouter H. Bos, Marieke M. ter Wee

**Affiliations:** 1 Amsterdam Rheumatology and Immunology Center, Reade, Admiraal Helfrichstraat 1, 1056 AA Amsterdam, The Netherlands; 2 Amsterdam Rheumatology and Immunology Center, Amsterdam UMC Location VUmc, Amsterdam, The Netherlands; 3 Department of Epidemiology and Data Science, Amsterdam UMC Location Vrije Universiteit, Boelelaan 1117, Amsterdam, The Netherlands; 4 Department of Rheumatology and Immunology, Amsterdam UMC Location Vrije Universiteit, Boelelaan 1117, Amsterdam, The Netherlands; 5 Amsterdam Public Health, Methodology, Societal Participation in Health, Amsterdam, The Netherlands; 6 Amsterdam Institute for Infection and Immunity, Inflammatory Diseases, Amsterdam, The Netherlands

**Keywords:** Inflammatory arthritis, Patient-reported outcomes, Electronic patient-reported outcomes, Adherence, Rheumatoid arthritis, Telemonitoring, eHealth, Telehealth, Facilitators, Barriers

## Abstract

Telemonitoring disease activity with electronic patient-reported outcomes (ePROs) may reduce the workload of rheumatic care by decreasing outpatient clinic visits. However, low adherence to reporting ePROs is frequently observed. Our objective was to identify facilitators and barriers to weekly monitoring of disease activity with ePROs. Patients with rheumatoid arthritis (RA), psoriatic arthritis (PsA), or ankylosing spondylitis (AS) who recently participated in one of the two telemonitoring studies with ePROs completed in a smartphone app, were invited to participate in focus group discussions (FGD). Thematic analysis was used to identify themes that play a role in the decision to continue or stop reporting weekly ePROs. A total of 22 patients participated in three FGDs. Five themes were identified that were of importance to adhere to telemonitoring: (1) questionnaire frequency, (2) discussing results of completed ePROs, (3) physical consultations, (4) patient insight into disease activity and (5) user experience of the app. All themes contained both barrier and facilitator elements. The results suggest that to improve adherence to telemonitoring of disease activity with ePROs, the perceived benefits of completing ePROs should be maximized. This can be done by providing patients the ability to skip (unneeded) physical consultations in case of low disease activity, and training clinicians to always discuss the completed ePROs. In addition, it is essential to reduce the effort to report ePROs, by tailoring the frequency of ePROs based on the patients’ disease activity or preference, aiming for optimal app functionality as well as by sending notifications when new ePROs are available.

**Supplementary Information:**

The online version contains supplementary material available at 10.1007/s00296-022-05263-5.

## Introduction

Regular quantification of disease activity is essential in the management of inflammatory arthritis (IA) [1, 2]. Measures to quantify disease activity are commonly composed of inflammation markers in the blood (erythrocyte sedimentation rate or *C*-reactive protein), physical examination (swollen and tender joint count), and patient-reported outcomes (PROs) [3, 4]. However, some measures that consist only out of PROs show high correlations with the composite measures and offer the advantage that patients can fill these out online at home (electronic PROs, ePROs) [5–7]. This facilitates new ways of monitoring disease activity, such as self-monitoring [8]. Here, patients regularly monitor their own disease activity in between consultations. Additionally, if the completed ePROs are connected to the electronic medical records at the rheumatology clinic, it gives the rheumatologist the opportunity to monitor disease activity over time, from a distance (telemonitoring). The adoption of these new techniques may be of great importance to optimize healthcare delivery and safe healthcare provider time spent per patient, as more efficiency will be necessary to deal with the combination of an increasing IA population and the estimated shortage of the rheumatology workforce.

Patients indicated in focus group discussions (FGDs) that apps used for monitoring disease activity with ePROs could be useful: they aid symptom management, provide insight into disease activity, make patients less dependent on healthcare providers, and could be used to allocate visits according to need [9–12]. However, ePRO self- and telemonitoring studies show that a substantial proportion of users stop reporting ePROs [13–15]. Recently, Seppen et al. reported that 53% of the ePROs were filled in during a 12 months randomized controlled telemonitoring trial [16]. Wiegel et al. [17] reported in a prospective cohort study that 31% of the participants stopped reporting ePROs after 10 weeks, and 64% after 26 weeks. This decline in adherence may reduce the potential benefits of telemonitoring disease activity. It is, therefore, essential to understand why patients stop reporting ePROs, so ways could be sought to improve long-term adherence.

So far, studies have shown that older patients and males may have higher odds to remain adherent to telemonitoring [14, 17]. However, due to the quantitative nature of these studies, the underlying reasons of why these associations are present, remain unknown. Most qualitative studies have focused on the perceived on user experiences, usability of the app used to collect ePROs, or the usability of ePROs itself, rather than the focus on facilitators and barriers to long-term adherence [8, 9, 12]. Only one qualitative study investigated the barriers to adherence of telemonitoring disease activity in 10 participants and found that barriers were mostly related to technical aspects [10]. Thus far, little is known about the incentives for patients to continue or stop monitoring their disease activity with ePROs over time. Therefore, the objective of this qualitative study is to investigate what the barriers and facilitators are to adhere to weekly monitoring of disease activity with ePROs in patients with inflammatory arthritis with the aim to identify factors that could be used to improve adherence.

## Methods

### The MyReumatism app

The app that was used by the participants to report the ePROs in both trials has been described elsewhere [8, 16]. In brief, the app sent out a weekly notification to complete a modified Multidimensional Health Assessment Questionnaire (mMDHAQ) that included the routine assessment of patient index data 3 (RAPID3) questionnaire in the app. Results were displayed in graphs over time to provide insight into disease activity over time. In addition, participants could access their medical data (lab results) in the app and in an information tab information was provided regarding rheumatic diseases and on the interpretation of the RAPID3.

### Focus group discussion

Participants were asked to participate in FGDs to identify the possible facilitators and barriers to adhere to monitoring of disease activity with ePROs. An FGD is a form of qualitative research that can be used to collect data on participant’s experiences, opinions and other more in-depth reasons behind a specific choice [18]. In each FGD, a maximum of 10 patients participate, and in advance, the structure of the FGDs were developed (Table 1) [19, 20].

**Table 1:** Focus group protocol

General introduction	Explanation of the structure and the aim of the FGD
Opening question	Could you introduce yourself and tell us since when you have been diagnosed with your rheumatic disease?
	How did you experience the use of the MyRheumatism App?
Follow-up question	What were reasons for you to continue or stop using the app during the study?
*Break*	
Follow-up question	What do you think of all the reasons listed for using or not using the app? Are there certain reasons missing?
	What would you need to optimally be able to monitor your rheumatic disease? Or what would you change to optimally monitor your rheumatic disease

*FGD* focus group discussion

### Data collection

Initially, the FGDs were planned in-person; however, due to COVID contact restrictions that were in place during the fall of 2021, the meetings were changed into online meetings. This change was made a week prior to the first FGD; participants were informed about this decision by telephone. The online FGDs were held with Zoom Video Communications (San Jose, United States) in December 2021. Participants received a link through which they could join the discussion in the browser or with an installed Zoom application.

Data on facilitators and barriers were gathered until saturation was reached. To reach data saturation, FGDs were performed until no new themes or explanations emerged from the participants [19, 20]. Data saturation was evaluated after every focus group by three researchers (MtW, BS, and JW), except the first.

Participants were told that the FGDs were moderated by a senior researcher with experience in qualitative research (MMtW). Two researchers who but did not engage in the discussion were present to take notes (JB and BS). During the FGD, cameras and microphones were continuously turned on to minimize impact of the online setting on the discussion. After 60 min, a 10-min break was held. Including the break, the FGDs lasted 100–120 min. The discussions were audiotaped and anonymously transcribed by an independent agency without personal data.

Prior to the FGD, participants were sent a brief questionnaire with 9 statements regarding self-monitoring. Participants were expected to indicate if they agreed with the statements on a scale ranging from 0 (do not agree at all) to 5 (totally agree). The statements were derived by WB, BS and JW from theoretical models aimed at explaining adherence. The statements and respective corresponding models are presented in the Supplementary Data Table S1.

### Focus group discussion sample

All participants that participated in the Self-Monitoring of Rheumatoid Arthritis (SEMORA) trial (*n* = 49) or the Digital Cohort of Inflammatory Rheumatic Diseases (DICODE) study (*n* = 220), were invited by email to participate in the FGDs. The main results of both trials have been published elsewhere [16, 17]. In short, both studies included patients over 18 years old, who were owner of a smartphone and able to read Dutch. The SEMORA study only included RA patients with a disease activity score 28 (DAS28) below 3.2 and excluded patients with DMARD treatment changes in the last 6 months. The DICODE study included RA, psoriatic arthritis (PSA) and ankylosing spondylitis (AS) patients, without additional requirements. In both studies, participants self-monitored their disease activity by completing a weekly ePRO in the MijnReuma Reade (MyRheumatism Reade) app. The SEMORA trial consisted of an intervention arm where self-monitoring was combined with patient-initiated care, and a control group where patients continued standard care. Only patients randomized to the intervention arm were invited to participate in the FGD’s. The DICODE study was a prospective cohort study where all patients were given the ability to self-monitor their disease activity as an add-on to usual care. No additional changes to their care were made. We needed a maximum of 10 participants for each FGD, independent of active users and drop-out. FGD participants were scheduled for the FGDs on a ‘first come first served’ basis.

### Data analysis

The primary aim was to uncover facilitators and barriers to weekly self-monitor disease activity with ePROs through an app. Thematic analysis was used to identify and interpret patterns of meaning across datasets [21]. Two researchers (BS and JW) read and analyzed the transcripts individually. Codes were given to the text based on its content. Relevant themes were identified and subsequently discussed. Ultimately, a set of major themes derived from the codes, was agreed upon during a consensus meeting between the two researchers. Any differences in views were resolved by discussion among the researchers. The software of Atlas.ti (Version 8.4., ATLAS.ti Scientific Software Development GmbH, Berlin, Germany) was used to code the transcripts and cluster the codes.

The consolidated criteria for reporting qualitative research (COREQ) checklist were used as guidance for the reporting of our qualitative research [22].

### Ethics

Verbal consent to participate in the FGDs was given and recorded by each participant prior to initiation of the discussion. This study was performed in compliance with the Declaration of Helsinki, local, national, ethical, and regulatory principles. This study is performed in accordance with relevant guidelines and regulations, and in specific the legislation of the medical ethics committee of the Vrije Universiteit medisch centum (VUmc) at Amsterdam, embedded in protocol number NL67704.029.19.

## Results

**Fig. 1:**
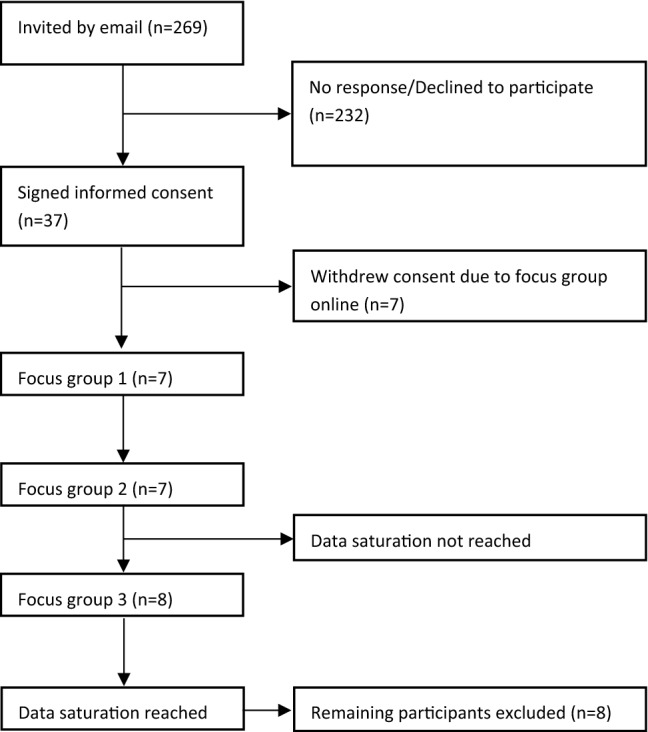
Patient flow diagram

In total, 269 patients who participated in either the DICODE or SEMORA study were approached to participate, of whom 37 agreed to participate in the focus group discussions. Of these 37 participants, seven withdrew their consent due to the change from in-person FGDs to online. So, a total of 30 patients were willing to participate in the focus groups. Initially, we started off with the first 22 patients (with seven, seven, and eight participants in each FGD, respectively), that were able to meet on our planned dates. During the third FGD, no new themes or explanations were heard, meaning data saturation was reached (see Fig. 1). Of the participants, eight had previously participated in the SEMORA trial and 14 in the DICODE trial. The mean age was 64 years (SD 10); 12 (55%) were women; 16 (73%) were diagnosed with RA, 4 (18%) with PsA, and 2 (9%) with AS. Mean RAPID3-scores of the participants at baseline of their respective study (SEMORA or DICODE) was 3.4 (SD 1.5), see Table 2.

**Table 2:** Participant characteristics

	Overall	FG1	FG2	FG3
Age, mean (SD)	64 (10)	68 (6)	60 (10)	62 (12)
Women, *n* (%)	12 (55)	4 (57)	4 (57)	4 (50)
*Diagnosis*				
RA, *n* (%)	16 (73)	5 (71)	3 (43)	8 (100)
PSA *n* (%)	4 (18)	1 (14)	3 (43)	0
AS *n* (%)	2 (9)	1 (14)	1 (14)	0
Disease duration, years	15 (12)	15 (11)	16 (15)	13 (12)
RAPID3 (SD)	3.4 (1.5)	3.7 (1.6)	3.4 (1.6)	3.1 (1.4)
Tertiary education, %	61	58	56	70
User activity ratio, % (min–max)	69	62 (12–100)	78 (35–100)	68 (15–100)

### Response to statements

In general, participants were positive about self-monitoring disease activity, see Table 3. However, participant responses were more evenly distributed between negative and positive regarding technical issues (question 6), the feeling that healthcare providers used the results (question 8), and frequency of the questionnaire (question 9).

**Table 3:**
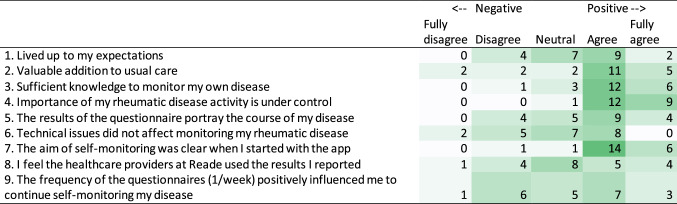
Response to the statements

The numbers represent how often the answer was given

### Thematic analysis

From the thematic analysis, five themes emerged that play a role in the adherence to completing ePROs. The five themes that emerged were: (1) questionnaire frequency, (2) discussing results of completed ePROs, (3) physical consultations, (4) patient disease activity insight, and (5) user experience of the app. All five themes contained elements that acted as a barrier and as a facilitator. The themes are discussed in this paper independently, and within each theme, we have specified the barrier and facilitator elements. Table 4 displays the quotes from participants corresponding to the aforementioned themes.

**Table 4:** Quotes and their corresponding theme

Quote number	Participant	Theme	Quote
1	2.4	Questionnaire frequency	I would say it is useful, completing a questionnaire every week, because my symptoms vary from week to week
2	2.7	Questionnaire frequency	Completing a questionnaire every week is not very useful to me, usually I do not feel much worse or better than last week
3	3.3	Questionnaire frequency	For people that have regular flares, yes, I would imagine that completing a questionnaire every week would be useful, however, in my case it has been stable for years, which uhm, makes its quite repetitive (laughs)
4	3.7	Questionnaire frequency	[…] that makes completing a questionnaire every week very tough, because, uhm, when you forget to complete it on your regular day, than the next time it will ask you to complete it on that day, and you have to make sure your questionnaires are up to date on a new day. At this moment, I would not know when the next one is due
5	1.10	Discussing results of completed ePROs	I noticed that all my data are also sent to the rheumatologist, and yes, that makes the conversation easier
6	2.2	Discussing results of completed ePROs	At a certain moment I remember thinking: why am I completing this? It felt like nobody was doing anything with the collected questionnaires. That made me think: why do I complete them? I never heard the doctor say something like ‘I have seen the results…’ or something like that
7	2.1	Discussing results of completed ePROs	However, I would be inclined to report the ePRO when I know that the rheumatologist reads the results, acts based on the results, and I can see in the app that the rheumatologist has seen the results
8	1.1	Physical consultations	I think reducing the number of consultations is a good aim. Especially because when my disease is stable and you must come in every 3–6 months, and you sit 10 min with your rheumatologist and he looks up and says: ‘well, blood is ok, do you have any symptoms? No? Ok, then I will see you next time’
9	3.8	Physical consultations	I was disappointed that the number of visits with my rheumatologist was reduced because I started using the app
10	3.8	Physical consultations	An important thing of consultations is that your joints are checked and that the rheumatologists looks if there is wear of the joints and so on. That could never be done in an app
11	2.1	Patient disease activity insight	When I visit my rheumatologist, then the app helps remembering: like, oh yeah right, that period my complaints worsened, but afterwards things improved
12	1.7	Patient disease activity insight	I can indicate how severe my symptoms were, but not where I had them (in which joints), which is information I would like to share with my rheumatologist
13	3.4	Patient disease activity insight	You do not want that all your symptoms are attributed to your rheumatic disease, while other things (diseases) may play a role. That makes completing the questionnaires difficult, which makes me think: I’ll skip this one, I do not want to make other people worried while these complaints have nothing to do with my rheumatic disease
14	1.2	User experience of the app	I used to get a notification that said my next questionnaire was to be completed. Now I do not get them and then you forget it
15	3.4	User experience of the app	The app had bugs, that’s why I stopped using it
16	1.10	User experience of the app	I did not mind spending 5 min of my time every week to complete a questionnaire

### Questionnaire frequency

The weekly questionnaire frequency was perceived as a facilitator to report more ePROs when disease activity was high or fluctuating (Q1). Also, the ability to make questionnaire completion a weekly habit facilitated participants to remember to report the ePRO. However, when disease activity was stable, the frequency was experienced too high and therefore a barrier (Q2). As the questionnaires became available exactly 1 week after completion, instead of the same day each week, some participants stated that forming a weekly habit was difficult, which was experienced as a barrier (Q4). Many participants indicated that the frequency of invitations to complete the questionnaire should be dependent on their disease activity (Q3).

### Discussing results of completed ePROs

Participants whose results of the completed ePROs were discussed, indicated that communication improved, and that they and their rheumatologists had a better understanding of their disease activity (Q5). This motivated and facilitated them to continue completing future questionnaires.

In contrast, some participants stated that their rheumatologist had never discussed the results. Therefore, they said, completion of the questionnaire felt pointless (Q6). These participants expressed that they would be motivated to report ePROs if rheumatologists would see the reported results and act on them (Q7).

### Physical consultations

In the current situation, patients have to visit the outpatient clinic, even when disease activity is stable or low. Participants stated that in those cases, physical appointments only provide limited value. For them, the ability to skip consultations if they monitored their disease activity with ePROs was a facilitator to keep reporting the weekly questionnaires (Q8). On the other hand, the fact that reporting ePROs could lead to a decrease in physical appointments made others hesitant to report ePROs. They were afraid that completing ePROs would replace their physical appointments. These participants stressed how important a physical appointment was to them (regardless of their disease activity status) (Q9). Furthermore, joint evaluations, and the consequent reassurance they were doing well, was highly appreciated by participants and they mentioned that these evaluations should not be replaced by scores in an app (Q10).

### Patient disease activity insight

By filling out the questionnaires, most participants gained insight in the development of their disease activity over time. This facilitated future reporting ePROs for these participants (Q11). However, this was not the case for all participants; some thought that the questionnaires were too general or lacked specificity. For them, not being able to give an accurate impression of their disease activity limited this (extra) insight and was a barrier to complete further questionnaires. For example, some participants wanted to exactly indicate which joints were tender or swollen each week (Q12) and wanted to provide context to the answers given in the questionnaire. Sometimes non-rheumatic symptoms led to a false increase in the RAPID3-score, which caused participants not to report the ePRO as they did not want to misinform their rheumatologist (Q13).

### User experience of the app

The functionality of the app and the interaction with the app were deemed important to participants. Various functionalities of the app acted as a facilitator to some and a barrier to others.

- Prompts
  Notifications (or reminders) did not work most of the time during both trials. Participants indicated that the prompt by notifications facilitated completing the ePROs greatly. They forgot to complete the questionnaire if no prompts were given (Q14).
- Bugs
  Several bugs occurred in the app during both trials which negatively impacted the user experience and was a barrier to future questionnaire completion (Q15). This was underlined as an important barrier for many participants (29 similar quotes).
- Effort
  It was important to participants that the effort needed to complete the questionnaire was low, this facilitated completion of the questionnaires (Q16).
- Desired features
  Participants expressed their desires for the option to include several extra features in the app that would increase the usability and perceived usefulness, and therefore could result in higher adherence. The most mentioned desired features were: (1) interacting with a healthcare provider through the app (mentioned 15 times); (2) monitoring rheumatic medication intake (including a ‘take medication notification’, mentioned 5 times), and (3) improving general health by monitoring weight, sport activities, or dietary advice (mentioned 5 times).

## Discussion

This study qualitatively evaluated facilitators and barriers linked to adherence for using an app to report ePROs in patients with inflammatory arthritis. The study revealed five themes that play a key role in the decision to stop or continue to report ePROs; the questionnaire frequency, discussing results of completed ePROs, physical consultations, patient insight into disease activity, and the user experience of the app. All five themes contained both facilitating and barrier elements.

In all FGDs, it stood out that experiencing at least one clear benefit of telemonitoring disease activity by reporting ePROs, was a major facilitator to continue to report ePROs. Although not all participants perceived all benefits, the most important benefits that facilitated patients to complete future ePROs were experiencing an improved insight into their own disease activity, the ability to skip physical consultations, and an improved communication with the rheumatologist when the completed ePROs were discussed (follow-up on completed ePROs). Consequently, a general perceived lack of benefits, most importantly when the completed ePROs were not discussed during the clinic visit, was a major barrier (as was highlighted by the results of statement 8). The benefits of telemonitoring disease activity with ePROs found in this study, were also found in similar qualitative studies in patients with IA. For example, the most important reason for using a telemonitoring program was the increased insight into patients’ own disease activity, as well as the ability to discuss the results with their rheumatologist [10, 23]. However, in these two studies, it was also reported that not all participants perceived monitoring disease activity with ePROs as an asset. Some participants indicated that they did not need an ePRO to gain insight into their disease activity, others expressed that during many consultations the results were not discussed, or that their symptoms were not captured well enough in the questionnaires, which ultimately led to not reporting the ePRO [10, 23]. Therefore, we think it is essential for the motivation of patients to continue to report ePROs, that at least one clear benefit is provided. This can be achieved by training healthcare providers to always discuss the completed ePROs during consultations, optimization of ePROs (making them more disease specific) and/or letting patients skip visits when their disease activity is low.

Providing a benefit gave participants an intrinsic incentive to continue completing ePROs. However, to adhere participants over time, our results suggest it is also essential to overcome barriers that negatively influences this incentive, and improve facilitators that help to maintain it. An important barrier that affected the incentive of participants was when reporting the ePRO was perceived as difficult or complicated, for example due to technical bugs, such as crashes of the app or the app not displaying the ePROs. This was also reflected in the quantitative data and also confirmed by another study [10]. Furthermore, participants indicated that the fixed interval of a weekly ePRO required a disproportionate effort when they had a low disease activity, which sometimes led to a discontinuation of weekly telemonitoring. On the other hand, participants also reported that making a habit out of completion facilitated adherence over a longer period to complete the ePROs. Therefore, we suggest implementing that patients can personalize their questionnaire frequency.

Another important contributor to the ability to complete ePROs was the notification when a new ePRO was available to be completed, as even motivated patients forgot to complete the ePROs when they were not reminded. All in all, ways should be sought to make reporting ePROs as simple and easy as possible. Therefore, we think that one should first aim to overcome and prevent bugs and optimize the user experience. Second, one should allow patients to tailor their ePRO frequency depending on their disease activity instead of following a fixed frequency. And at last, we advise to implement notifications when new ePROs are available.

### Strengths and limitations

With our qualitative methods, we were able to examine the underlying barriers and facilitators to reporting ePROs. This resulted in a thorough evaluation and description of themes, and subsequently recommendations to increase adherence. In addition, our results are in line with most of the EULAR points to consider, particularly regarding tailoring to patient preference (although our advice is ePRO frequency specific), the (equipment) and training of the healthcare team, the use for disease monitoring. We demonstrates agreement between—foremostly—physicians (EULAR ptc) and patients. A limitation of this study was that selection bias may have occurred, which could influence the generalizability. Selection bias is often present in telemonitoring studies in rheumatology, and is also likely present in this study as the participants were a subset of previously performed telemonitoring studies, that both included a relatively high percentage of patients with tertiary education [5, 16, 17]. This is highlighted by the seven patients who did not want to participate after changing the FGD to online. Therefore, the results of this study, as well as in both trials, lack the data and opinions of the patients who do not want to participate in a telemonitoring study or use telemonitoring at all. In addition, a small sample of AS and PSA patients were included in the study, limiting the generalizability to the IA population. However, the second focus group consisted for more than 50% out of patients with AS and PsA, and did not result in other themes or differences between mentioned themes by the two diagnosis groups. We, therefore, think that it is likely that reasons to stop and continue telemonitoring would be similar for all diagnosis groups with IA.

Second, performing FGDs online is relatively new and could possibly alter the group-dynamic and discussion when compared to on-site FGDs. However, recent studies have shown that online FGDs are well accepted by patients and generate similar content when given face to face [24–27].

## Conclusion

The results suggest that to improve adherence to telemonitoring of disease activity with ePROs, the perceived benefits of completing ePROs should be maximized. This can be done by providing patients the ability to skip (unneeded) physical consultations in case of low disease activity, and training clinicians to always discuss the completed ePROs. In addition, it is also essential to reduce the effort to report ePROs, by tailoring the frequency of ePROs to their disease activity or the patient’s preference, aiming for optimal app functionality as well as by sending notifications when new ePROs are available.

## Supplementary Information

Below is the link to the electronic supplementary material.

Supplementary file 1 (DOCX 14 kb)

## Data Availability

Data are available on request due to privacy or other restrictions.
