# Supplementary material for: Facilitators and barriers to adhere to monitoring disease activity with ePROs: a focus group study in patients with inflammatory arthritis
Source: Rheumatol Int. 2023 Jan 10;43(4):677–85. doi: 10.1007/s00296-022-05263-5 (PMC9995401; doi:10.1007/s00296-022-05263-5)
Supplement: Supplementary file 1 — (DOCX 14 kb) [file 296_2022_5263_MOESM1_ESM.docx]

# Supplement 1

Supplementary Table S1. Statements and the theoretical model of which they are derived.

| **Statements** | **Theoretical model** |
| --- | --- |
| 1. Self-monitoring my disease activity lived up to my expectations | Supportive Accountability(1) |
| 2. Self-monitoring was a valuable addition to usual care | Health Belief Model(2) |
| 3. I had sufficient knowledge to monitor my own disease | Theoretical Domains Framework(3) |
| 4. It is important that my rheumatic disease activity is under control | Health Belief Model(2) |
| 5. The results of the weekly questionnaire portray the course of my disease | Technology Acceptance Model(4) |
| 6. Technical issues did not affected the self-monitoring of my rheumatic disease | Technology Acceptance Model(4) |
| 7. The purpose of self-monitoring was clear when I started with the app | Supportive Accountability (1) |
| 8. I feel the healthcare providers at Reade use the results of self-monitoring | Supportive Accountability (1) |
| 9. The frequency of the questionnaires (1/week) positively influenced me to continue self-monitoring my disease | Health Belief Model(2) |
|  |  |

References

1. Mohr DC, Cuijpers P, Lehman K. Supportive accountability: a model for providing human support to enhance adherence to eHealth interventions. Journal of medical Internet research. 2011;13(1):e30.

2. Janz NK, Becker MH. The Health Belief Model: A Decade Later. Health Education Quarterly. 1984;11(1):1-47.

3. Cane J, O’Connor D, Michie S. Validation of the theoretical domains framework for use in behaviour change and implementation research. Implementation Science. 2012;7(1):37.

4. Holden RJ, Karsh BT. The technology acceptance model: its past and its future in health care. Journal of biomedical informatics. 2010;43(1):159-72.
